# Morphological and Molecular Characterization of Reared Parasitoid Wasps of the Genus *Glyptapanteles* Ashmead 1904 (Insecta: Hymenoptera: Braconidae: Microgastrinae) Associated with Lepidoptera in India

**DOI:** 10.1371/journal.pone.0150765

**Published:** 2016-03-04

**Authors:** Ankita Gupta, Thiruvengadam Venkatesan, Ravi P. More

**Affiliations:** ICAR-National Bureau of Agricultural Insect Resources, Post Bag No. 2491, H. A. Farm Post, Bellary Road, Hebbal, Bangalore, 560 024, Karnataka, India; University of Innsbruck, AUSTRIA

## Abstract

*Glyptapanteles* Ashmead (Hymenoptera: Braconidae: Microgastrinae) is a cosmopolitan group of hyperdiverse parasitic wasps. The genus remains taxonomically challenging in India due to its highly speciose nature, morphological similarity amongst species and negligible host records. The Indian fauna is one of the most diverse and also the least studied. The present study is based on 60 populations reared from 35 host species, 100+ individual caterpillar rearings (1100 wasp specimens pinned and 2000 in alcohol) and from 12 different geographical locations of the country (11 states and one Union territory) that represent 26 provisional *Glyptapanteles* species within 8 species-groups. Out of 60 populations, phylogenetic analyses were performed on 38 based on mitochondrial cytochrome oxidase subunit I (COI) nucleotide sequences. Maximum likelihood and Bayesian inference methods displayed three and four major discrete *Glyptapanteles* clades, respectively. In clade A very few Indian species were grouped along with Neotropical and Thailand species. The other clades B and C grouped the majority of the Indian species and showed considerable host specificity in both the trees. All parasitic wasp species were gregarious in nature, except for two populations. Three different sets of data (morphology, host records, and COI) were integrated in order to generate accurate boundaries between species/species-groups. Illustrations of all parasitized caterpillars/cocoons and 42 habitus views of *Glyptapanteles* spp., distributional information, and GenBank accession numbers, are presented. The present study, perhaps the most comprehensive done to date in India, suggests the presence of several additional *Glyptapanteles* species, which were previously unrecognized.

## Introduction

Microgastrine wasps are important in biological control because they parasitize caterpillars of many families of Lepidoptera. The species of the genus *Glyptapanteles* Ashmead 1904 (Hymenoptera: Braconidae: Microgastrinae) are hyperdiverse parasitic wasps with cosmopolitan distribution. They are parasitic in immature stage and free-living as adults. So far, 122 species have been described worldwide [1–4].

*Glyptapanteles* is taxonomically challenging because they are morphologically homogeneous and highly speciose [5]. Recently, a classical paper reviewing genus *Apanteles sensu stricto* followed the combined approach including traditional taxonomy, molecular, software-based, biology, and geography to facilitate the process of finding and describing new species in a hyperdiverse group such as *Apanteles* [6]. A similar approach is followed for the genus *Glyptapanteles* [7].

**Fig 1:**
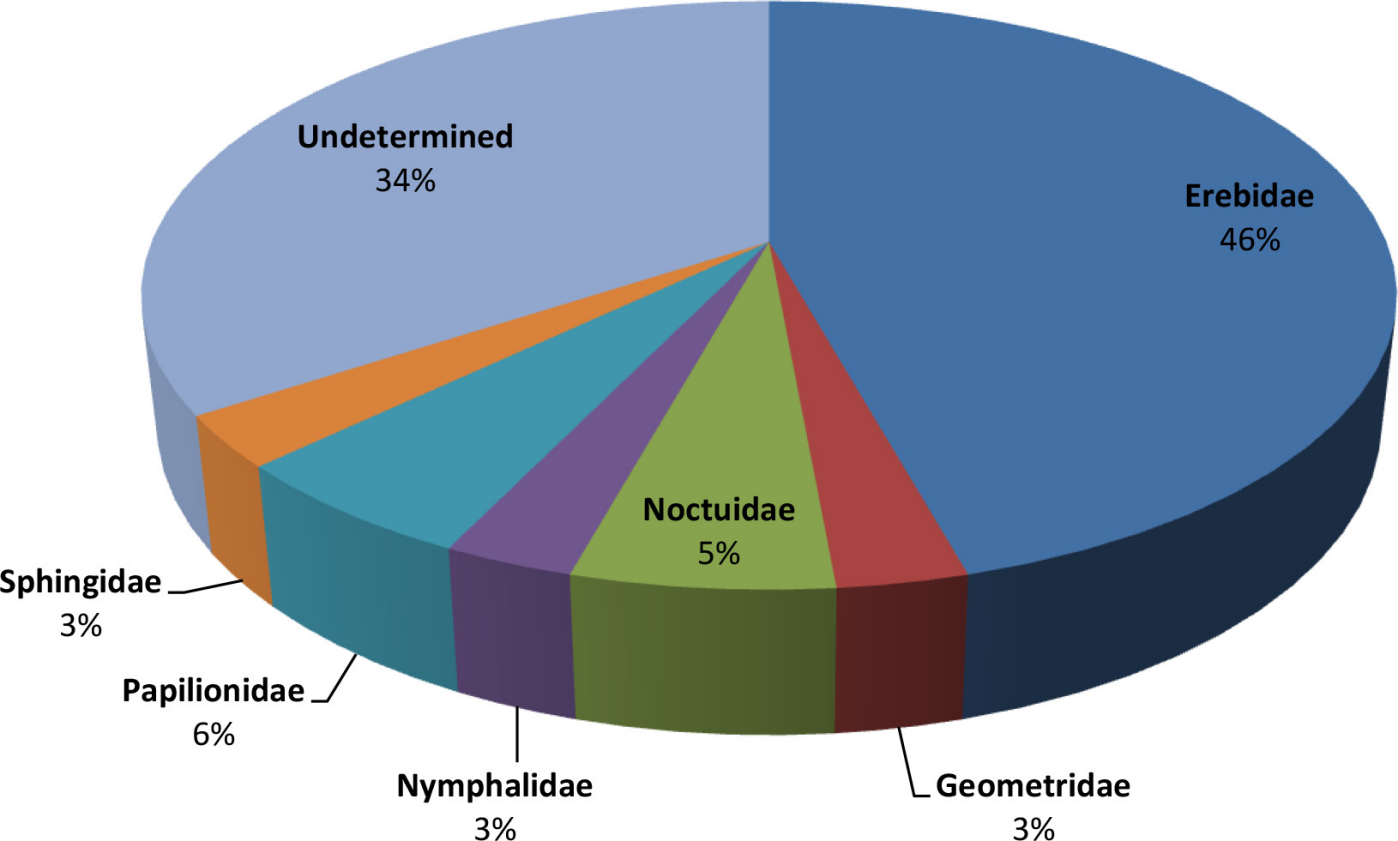
Proportion of Lepidoptera families parasitized by Indian *Glyptapanteles* species from India.

Many families of the order Lepidoptera have been reported as the hosts for the genus *Glyptapanteles* [8, 9] (Table 1). The proportion of Lepidoptera families parasitized by Indian *Glyptapanteles* species with known host records from India is presented in Fig 1.

**Table 1:** Details of host families documented for the genus *Glyptapanteles*.

S. No.	Host families	Geographical region	Reference
1.	Apatelodidae, Arctiidae, Geometridae, Limacodidae*, Noctuidae, Nymphalidae, Pieridae, Pyralidae and Saturniidae	Neotropical region (Ecuador)	Whitfield et al. 2009 [8]
2.	Arctiidae, Bombycidae, Crambidae, Elaschidae, Geometridae, Hesperiidae, Limacodidae, Lycaenidae, Noctuidae, Notodontidae, Nymphalidae, Pieridae, Riodinidae, Saturnidae, Sphingidae, and Tortricidae	Neotropical region (northwestern Costa Rica)	Arias-Penna 2011[9]
3.	Erebidae (Arctiinae; Lymantriinae), Geometridae, Noctuidae (Noctuinae), Nymphalidae, Papilionidae* and Sphingidae	Oriental region (India)	Present study

* Unusual record

In the present study, to decide on accurate boundaries between the species/species-groups, external morphological characters with high magnification images, associated hosts and mitochondrial gene cytochrome *c* oxidase (COI) have been employed because COI gene of the mitochondrial DNA have commonly been widely referred as molecular marker. As already mentioned, the aim of this paper is to focus on species delimitation, host specificity and to infer phylogenetic relationships, if any. Phylogenetic reconstructions based on the COI gene of *Glyptapanteles* spp. using Maximum Likelihood (ML) and Bayesian analyses were performed and significant difference in the degree of host specificity combining morphological data was observed in some Indian populations. Altogether about 600–645 base pairs were analyzed from sequences obtained from COI for 38 morphospecies of *Glyptapanteles* reared from 35 morphospecies of lepidopteran hosts from the Indian region.

## Material and Methods

### 1. Collection of samples

The collection localities covered 11 States and one Union Territory of India. Collections were not done from any national park or other protected area of land or sea, or on any private land, hence no permission was required. No specific permissions were required for any of the collection localities/activities, as the collections were done in and around ICAR research Institutes as NBAIR acts as a government nodal agency for collection, characterization, documentation, conservation, exchange, and utilization of agriculturally important insect resources (including mites, spiders and related arthropods) for sustainable agriculture in India. The field studies did not involve any endangered or protected species.

Altogether, 3,000+ specimens from 100+ individual caterpillar rearings and 88 COI nucleotide sequences of *Glyptapanteles* spp. were studied. Caterpillars were handpicked from various crop fields, forest trees, and wild plantation during 2010–2015. Caterpillars from the same host plant were kept separately in individual plastic containers with fine meshed lids carrying specific code numbers in the laboratory. Unparasitized caterpillars from the same brood were reared to adulthood for host confirmation. Caterpillar rearings were conducted at 25±2°C and 50±10% relative humidity and were fed with the natural host plant tissue from which the respective caterpillar was collected. More than one hundred caterpillars were reared individually, and ~3000 microgastrine wasps were recovered. The wasps were later curated, mounted and taxonomically examined. The caterpillars/cocoons were primarily coded based on the date of collection, host plant and locality. Wasp specimens were reared from field collected caterpillars, as well as recovered from field collected cocoons. The specimen codes for the taxonomic and molecular treatment remain the same. Sequences of each population have been deposited in GenBank (www.ncbi.nlm.nih.gov).

The collection of ICAR-NBAIR Museum, Commonwealth Institute of Biological Control (CIBC)-Indian Station-Bangalore, and the National Pusa Collection (NPC), Indian Agricultural Research Institute, New Delhi were examined. Additionally, types from the Natural History Museum, London, UK (BMNH) housing collections of *Glyptapanteles* from South East Asia were examined. Identification was undertaken consulting Wilkinson 1928 [10], Nixon 1965 [11], Mason 1981 [3], Gupta & Pereira 2012 [12], Gupta 2013 [13], Gupta & Fernández-Triana 2014 [14] and Yu et al. 2012 [15] was consulted for host associations. Most of the images of host caterpillar/cocoons were taken with either Nikon D7000 with Nikkor 105mm macro lens or Sony DSLR-A100 Digital SLR Camera with Tamron 90mm macro lens. The wasp images were taken using Leica M 205 A Stereozoom microscope with Leica DC 420 inbuilt camera using Automontage software (version 3.8). All specimens of the present study are housed in the ICAR-National Bureau of Agricultural Insect Resources (NBAIR), Bangalore, India.

### 2. DNA extraction and sequencing

#### 2.1. DNA extraction and PCR amplification

Each species of *Glyptapanteles* adults was separately preserved in 95% ethanol at -20°C and used for DNA extraction. The adults were thoroughly washed with formaldehyde and alcohol to avoid contamination of any other DNA. The genomic DNA was isolated using a DNA extraction kit (QIAGEN DNeasy blood and tissue kit Cat. 69504, Germany). Polymerase chain reactions involved the use of 2μl DNA template, 3μl (10x) Taq assay buffer, 1μl dNTPs (each in 10 mM concentration), 1.5μl forward and reverse primers (10 picomoles/μl), 1μl Taq Polymerase (1 U) and the final PCR mixture made up to 30 μl in distilled sterile water. The primers used to amplify the CO-1 region were LCO1490 5’-GGTCAACAAATCATAAAGATATTGG-3’ (forward) and HCO2198 5’-TAAACTTCAGGGTGACCAAAAAATCA-3’ (reverse) [16]. Amplification involved a thermal cycler (c-1000 Thermal Cycler, Biorad Laboratories, California) programmed to 95°C for 5 minutes, followed by 34 cycles of 94°C for 30 seconds, 53°C for 30 seconds and 72°C for one minute and a final extension at 72°C for 10 minutes. Amplified products were separated on 1.2% agarose (ACROS). Gels were stained using ethidium bromide. Molecular standards were run along with the samples for reference. The PCR products were gel eluted with QIAquick Gel Extraction Kit (Cat. No. 28704, Germany) and direct sequenced through Applied Biosystems ABI prism 310. Nuclear copies of mitochondrial DNA (NUMT) contamination were safeguarded by performing amino acid translation by checking for the stop codons in the sequence.

#### 2.2. DNA sequence analyses

Sequence chromatograms of forward and reverse READS were assembled and edited using CLC Genomics Workbench 7. The similarity search of resulting consensus sequences was performed using Basic Local Alignment Search Tool (BLAST) against sequences in GenBank to confirm that the sequence was indeed corresponding taxonomy. All generated COI consensus sequences have been deposited in NCBI GenBank database [17]. The accession numbers of all specimens for GenBank are mentioned in Table 2.

**Table 2:** Showing GenBank Accession Numbers, specimen code along with locality data, wasp species, associated host information along with images of host and parasitoid in habitat.

S. No.	GenBank Accession No.	Specimen Code and locality	Wasp species	Host species
1.	KR260984	14810RH Karnataka	*Glyptapanteles* sp.	*Lymantria* sp. (Erebidae, Lymantriinae) on *Achras sapota*/*Manilkara zapota*
2.	KT254313	5711Andhra Pradesh	*Glyptapanteles* sp.	*Utetheisa pulchelloides* Hampson, (Erebidae: Arctiinae) on *Crotalaria juncea* L.
3.	KT284335	301014AKarnataka: Chintamani	*Glyptapanteles* sp.	Indeterminate caterpillar on *Cajanus cajan* (L.) Millsp.
4.	KR260983	10312 Andaman Island: Wandoor beach	*Glyptapanteles* cf. *spodopterae* Ahmad	? *Melanephia mosara* Swinhoe (Noctuidae) on *Ipomoea pes-caprae* (L.) R. Br.
5.	KR260976	1014SL Karnataka: Bangalore	*Glyptapanteles spodopterae*Ahmad	*Spodoptera litura* (Fab.) (Noctuidae)
6.	KT25318	19714BO Karnataka: Savandurga	*Glyptapanteles* sp.	Indeterminate caterpillar (Erebidae:? Arctiinae)
7.	KR260978	51112 Karnataka: Bangalore	*Glyptapanteles* (= *Protapanteles*) cf. *colemani* (Viereck)	*Orgyia postica* Walker (Erebidae: Lymantriinae) on *Cajanus cajan* (L.) Millsp.
8.	KR260982	51005GKarnataka: Bangalore	*Glyptapanteles* (= *Cotesia*) cf. *bataviensis* (Rohwer)	Indeterminate caterpillar (Erebidae: Arctiinae) on grass
9.	KT284337	311014AKarnataka: Chintamani	*Glyptapanteles* sp.	Not known
10.	KT284338	81114ADAssam	*Glyptapanteles* (= *Protapanteles*) cf. *artonae* (Rohwer)	Not known
11.	KT254315	200813P Karnataka: Bangalore	*Glyptapanteles aristolochiae* (Wilkinson)	*Pachliopta hector* (Linnaeus) (Papilionidae) on *Aristolochia indica* L.
12.	KT254317	41013 West Bengal: Nandigrama	*Glyptapanteles* cf. *lamprosemae* (Wilkinson)	Not known
13.	KT284344	111011Maharashtra: Mumbai	*Glyptapanteles hypermnestrae* Gupta & Pereira	*Elymnias hypermnestra* (Linnaeus) Nymphalidae on *Cocus nucifera* L.
14.	KR021157	221111 Karnataka: Kalyan Nagar	*Clanis phalaris* Gupta	*Clanis phalaris* Cramer (Sphingidae) on *Pongamia pinnata* (L.)
15.	KT254311	19911Karnataka: Vijayapura	*Glyptapanteles* cf. *lamprosemae* (Ahmad)	Indeterminate caterpillar (Erebidae: Arctiinae) on*Syzygium cumini* (L.)
16.	KT284334	3914Karnataka: Kailasagiri	*Glyptapanteles* sp.	Not known
17.	KT284343	5714A2 Tamil Nadu: Valparai	*Glyptapanteles* sp.	*Nepita conferta* (= *Asura conferta*) (Erebidae: Arctiinae)
18.	KR021155	14912 Karnataka: Bidar	*Glyptapanteles* sp.	*Ascotis imparata* (Walker) (Geometridae) on *Melia azedarach* L.
19.	KT254321	3714 Tamil Nadu: Valparai	*Glyptapanteles* sp.	*Nepita conferta* (= *Asura conferta*) (Erebidae: Arctiinae)
20.	KR021152	271212Karnataka: Yugavana Hills	*Glyptapanteles obliquae* (Wilkinson)	Indeterminate caterpillar (Erebidae: Arctiinae) on *Nerium* sp.
21.	KR021153	20712 Tamil Nadu: Bagalur	*Glyptapanteles obliquae* (Wilkinson)	*Spilosoma obliqua* (Walker) (Erebidae: Arctiinae)
22.	KT254320	13314 Karnataka: Mudigere	*Glyptapanteles* cf. *obliquae* var. *niger* (Wilkinson)	Indeterminate caterpillar (Erebidae: Arctiinae)
23.	KR021160	2714A4 Tamil Nadu: Valparai	*Glyptapanteles* sp.	*Nepita conferta* (= *Asura conferta*) Erebidae: Arctiinae
24.	KR021154	101012 Karnataka: Kanakapura	*Glyptapanteles creatonoti* (Viereck)	Indeterminate Erebidae: Arctiinae
25.	KT254319	24114Pu Kerala: Pattambi	*Glyptapanteles* sp.	Not known
26.	KT254316	201205 Karnataka: Vaddarahalli	*Glyptapanteles* sp.	Not known
27.	KT284339	190913ab Karnataka: Hessaraghatta	*Glyptapanteles* sp.	Not known
28.	KT254310	2810Karnataka: Savandurga	*Glyptapanteles* cf. *caberae* (Marshall)	Not known
29.	KT284342	25315 Goa: Mollem	*Glyptapanteles* cf. *lamprosemae* (Ahmad)	Not known
30.	KP153535	12912 Karnataka: Bidar	*Glyptapanteles* (= *Protapanteles*) cf. *acherontiae* (Cameron)	Undetermined caterpillar on *Azadirachta indica*A. Juss
31.	KR260981	16810SA Karnataka: Sadanapalya	*Glyptapanteles* (= *Protapanteles*) cf. *acherontiae* (Cameron)	Undetermined caterpillar on *Azadirachta indica*A. Juss
32.	KR260977	14912U Uttarakhand: Almora	*Glyptapanteles* (= *Protapanteles*) cf. *acherontiae* (Cameron)	Not known
33.	KR260980	SED60R Karnataka: Gulburga	*Glyptapanteles* cf. *creatonoti* (Viereck)	*Argina astrea* (Drury) (Erebidae: Arctiinae) on *Crotolaria juncea* L.
34.	KR021163	41114 Sikkim	*Glyptapanteles* (= *Protapanteles*) cf. *acherontiae* (Cameron)	Not known
35.	KR021156	18413Tamil Nadu: Kotagiri	*Glyptapanteles* cf. *aristolochiae* (Wilkinson)	Not known
36.	KR021159	100113 Karnataka: Bangalore	*Glyptapanteles aristolochiae* (Wilkinson)	*Pachliopta hector* (L.) (Papilionidae)
37.	KR021162	SAJK13 Kerala: Kozhikode	*Glyptapanteles aristolochiae* (Wilkinson)	*Troides minos* Cramer (Papilionidae)
38.	KR021151	101211Karnataka: Chikkaballapur	*Glyptapanteles* sp.	(Erebidae: Arctiinae)
39.	KT254314	CA2711Karnataka: Savandurga	*Glyptapanteles* sp.	Not known
40.	Yet to receive	7715HR Tamil Nadu: Hosur	*Glyptapanteles* (= *Cotesia*) cf. *bataviensis* (Rohwer)	Indeterminate caterpillar (Erebidae: Orgyiini) on *Parthenium* sp.
41.	KU258053	23914D Himachal Pradesh: Dalhousie	*Glyptapanteles* sp.	Not known
42.	Yet to receive	3815CA Karnataka: Kawalibaisandra	*Glyptapanteles* sp.	Indeterminate caterpillar on *Annona squamosa* L.

#### 2.3. Phylogenetic analyses

For phylogenetic analysis, the homologous COI sequences from Genbank database (NCBI) were obtained by performing similarity searches using BLAST tool [18]. The top hits (sequences) with high similarity score and E-values equal to zero were considered, and only non-redundant species sequences were retained for further analysis (S1 Table). In our COI dataset, altogether 88 gene sequences were chosen, which were distributed into 38 Indian sequences and 50 BLAST sequence hits. A parasitoid wasp, *Parapanteles*, from another genus of the same subfamily (Microgastrinae, tribe Cotesiini) was included as outgroup in sequence analyses. All 89 COI sequences were aligned using MUSCLE multiple alignment program with the default alignment parameters [19]. The variable sites analyses from the alignment of the dataset were performed in MEGA 6.0 [20].

Bayesian inference (BI) and Maximum Likelihood (ML) methods were used to reconstruct phylogenetic trees of *Glyptapanteles*. For BI and ML methods, model selection was based on the Akaike information criterion (AIC) computed by PartitionFinder version 1.1.1 software [21]. The subset partitions with positions 1, 2 and 3 were done and the best fit substitution models were predicted. The BI analyses was performed using MrBayes version 3.2.2 [22], a stop rule convergence value of 0.01 was set and a stoprule placed on the run when that value was reached, which occurred on the 1140000 Markov chain Monte Carlo (MCMC) generation and two incrementally heated chains. MCMC started from a random tree and sampling one of every 500 generations, with the first 570 (25%) of the trees discarded as burn-in out of 2280 trees. The remaining trees were used to create a majority-rule consensus tree. Maximum Likelihood tree search was performed in RAxML version 7.0.4 software using the predicted model and bootstrap support values for each node in the ML tree was calculated with 1000 searches [23]. We used the 'ML+ Rapid Bootstrap' option to perform analysis. The resulting Maximum Likelihood and Bayesian inference phylogenetic trees were imported, edited and visualised in the FigTree software version 1.4.2 (http://tree.bio.ed.ac.uk/software/figtree/).

#### 2.4. Intra- and Inter- specific evolutionary divergence

Pairwise Kimura-2-parameter (K2P) distance was measured to understand the evolutionary divergence rate among the species based on COI gene [24]. We used the datasets which were used for the phylogenetic tree analyses excluding outgroup and estimated the pairwise intra- and inter- species K2P distances among the species sequences using option implemented in MEGA version 6.0 software [20]. K2P distances of our interested species sequence pairs were obtained.

The following acronyms are used:

**BMNH:** The Natural History Museum, London, United Kingdom

**ICAR:** Indian Council of Agricultural Research, New Delhi, India

**NBAIR**-National Bureau of Agricultural Insect Resources, Bangalore, India

## Results

### Genus *Glyptapanteles* Ashmead

#### 3.1. Brief diagnosis

**Fig 2:**
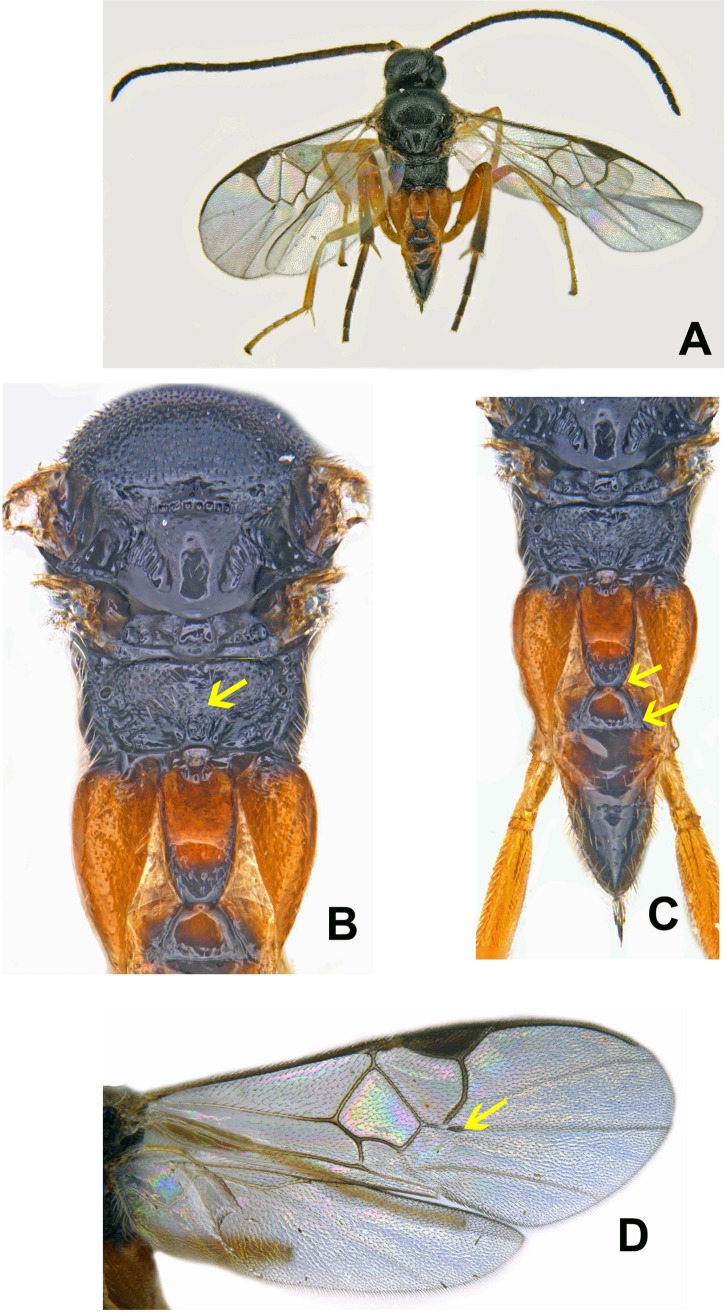
Automontage images of *Glyptapanteles creatonoti* (Viereck). Female in dorsal view (A), mesosoma with metasoma in part (B) metasoma along with mesosoma in part (C), wings (D).

*Glyptapanteles* can be distinguished from other microgastrine genera by the following combination of features (see Fig 2A–2D): forewing with second r-m vein absent, small areolet is open distally (Fig 2D); propodeum coarsely sculptured with medial carina rather than medial areola, or nothing medially (Fig 2B); first metasomal tergite narrowing posteriorly, second metasomal tergite broadening posteriorly and often nearly triangular (Fig 2C); ovipositor and sheaths short and barely exserted. The genus is distinctive, although it could be confused with some *Distatrix* [8].

#### 3.2. Host association

**Fig 3:**
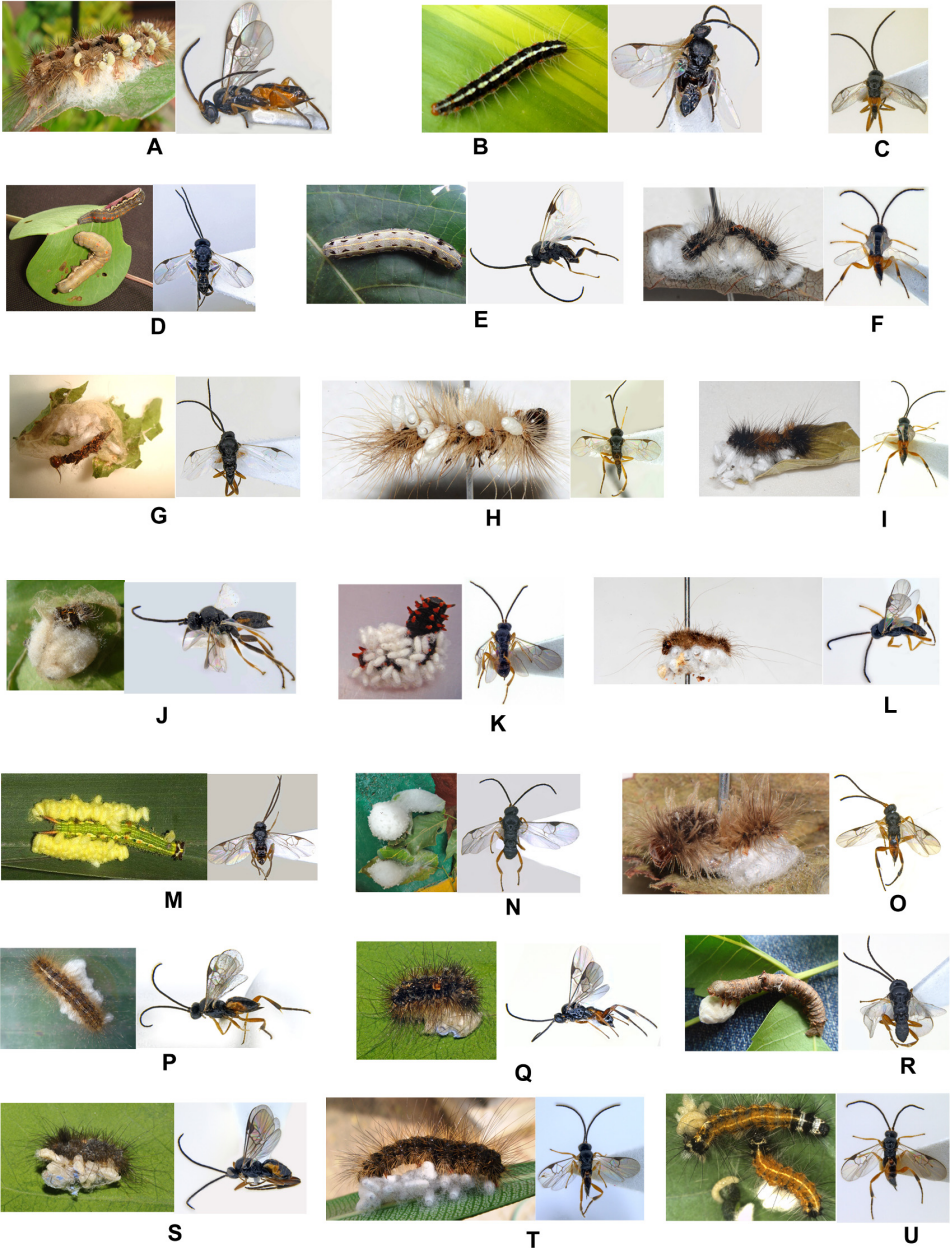
Host and associated *Glyptapanteles* species. *Lymantria* sp. & *Glyptapanteles* sp. 14810RH (A), *Utetheisa pulchelloides* & *Glyptapanteles* sp. 5711 (B), *Glyptapanteles* sp. 301014A (C),? *Melanephia mosara* & *G*. cf. *spodopterae* 10312 (D), *Spodoptera litura* & *G*. *spodopterae* 1014SL (E), indet. host & *Glyptapanteles* sp. 19714BO (F), *Orgyia postica* & *G*. cf. *colemani* 51112 (G), indet. host & *G*. cf. *bataviensis* 51005G (H), indet. host & *Glyptapanteles* sp. 311014A (I), indet. host & *G*. cf. *artonae* 81114AD (J), *Pachliopta hector* & *G*. *aristolochiae* 200813P (K), indet. host & *G*. cf. *lamprosemae* 41013 (L), *Elymnias hypermnestra* & *G*. *hypermnestrae* 111011 (M), *Clanis phalaris* & *G*. *clanisae* 221111 (N), indet. host & *G*. cf. *lamprosemae* 19911 (O), indet. host & *Glyptapanteles* sp. 3914 (P), *Nepita conferta* & *Glyptapanteles* sp. 5714A2 (Q), *Ascotis imparata* & *Glyptapanteles* sp. 14912 (R), *Nepita conferta* & *Glyptapanteles* sp. 3714 (S), indet. host & *G*. *obliquae* 271212 (T), *Spilosoma obliqua* & *G*. *obliquae* 20712 (U).

**Fig 4:**
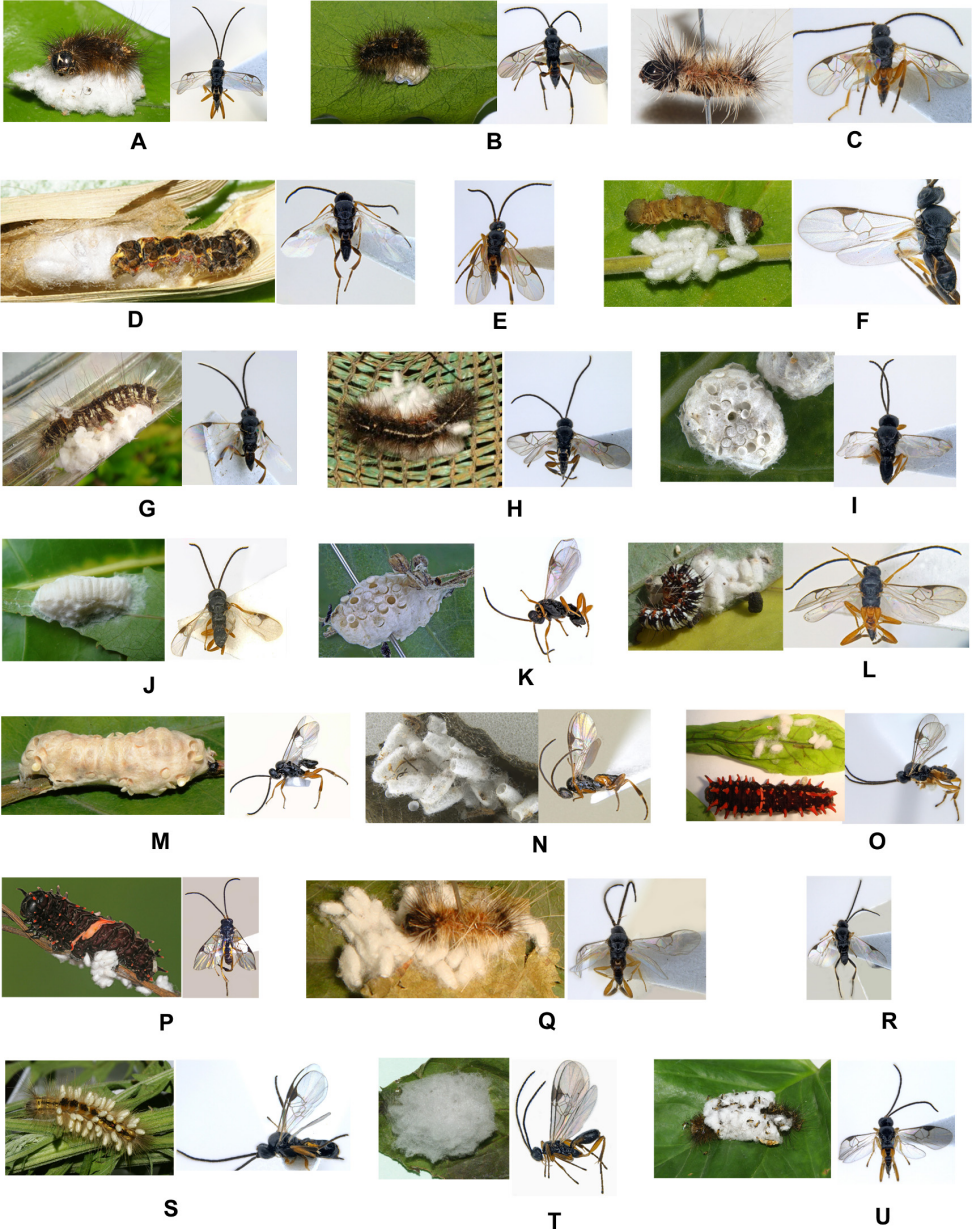
Host and associated *Glyptapanteles* species. Indet. host & *G*. cf. *obliquae* var. *niger* 13314 (A), *Nepita conferta* & *Glyptapanteles* sp. 2714A4 (B), indet. host & *G*. *creatonoti* 101012 (C), indet. host & *Glyptapanteles* sp. 24114Pu (D), *Glyptapanteles* sp. 201205 (E), indet. host & *Glyptapanteles* sp. 190913ab (F), indet. host & *G*. cf. *caberae* 2810 (G), indet. host & *G*. cf. *lamprosemae* 25315 (H), cocoon & *Glyptapanteles* cf. *acherontiae* 12912 (I), cocoon & *G*. cf. *acherontiae* 16810SA (J), cocoon & *G*. cf. *acherontiae* 14912U wasp (K), *Argina astrea* & *G*. cf. *creatonoti* SED60R (L), cocoon & *G*. cf. *acherontiae* 41114 (M), cocoon & *G*. cf. *aristolochiae* 18413 (N), *Pachliopta hector* & *G*. *aristolochiae* 100113 (O), *Troides minos* & *G*. *aristolochiae* SAJK13 (P), indet. host & *Glyptapanteles* sp. 101211 (Q), *Glyptapanteles* sp. CA2711 (R), indet. host & *G*. cf. *bataviensis* 7715HR (S), cocoon & *Glyptapanteles* sp. 23914D (T), indet. host & *Glyptapanteles* sp. 3815CA (U).

First comprehensive documentation of Lepidoptera families/subfamilies reported as hosts of *Glyptapanteles* spp. from India includes: Erebidae (Arctiinae; Lymantriinae), Geometridae, Noctuidae (Noctuinae), Nymphalidae, Papilionidae and Sphingidae. All parasitic wasp species were gregarious in nature baring two populations- *Glyptapanteles spodopterae* Ahmad reared from *Spodoptera litura* (Fab.) from mainland India and *Glyptapanteles* cf. *spodopterae* Ahmad from? *Melanephia mosara* Swinhoe associated with *Ipomoea pes-caprae* (L.) R. Br. from Andamans Islands. Also many interesting host records are documented for the first time from India- *Glyptapanteles aristolochiae* (Wilkinson) from caterpillar of *Troides minos* (Cramer) (Papilionidae); *Glyptapanteles* spp. from varied hosts- *Lymantria* sp. (Erebidae), *Utetheisa pulchelloides* (L.) (Erebidae), *Orgyia postica* Walker (Erebidae), *Nepita* (= *Asura*) *conferta* (Walker) (Erebidae), *Ascotis imparata* (Walker) (Geometridae), and *Argina astrea* (Drury) (Erebidae). (Fig 3A–3U) & (Fig 4A–4U) represent all hosts and associated parasitoids along with the code numbers mentioned in Table 2.

#### 3.3. Phylogenetic analyses

About 600–645 bp were sequenced from mtDNA COI for 38 morphospecies of *Glyptapanteles* reared from 35 morphospecies of lepidopteran hosts from the Indian region. The alignment of the COI dataset resulted in a total of 701 nucleotide sites, of which 279 were variable sites. Selecting a suitable partitioning scheme is an essential step in most analyses because it can influence the accurateness of phylogenetic reconstruction. This is in agreement with the findings of Whitefild et al. [25], according to which the general time reversible (GTR) substitution model was referred in ML and BI methods to study the microgastrine braconid wasp genera. Therefore, best-fit partitioning schemes and nucleotide substitution models were chosen. In the ML analysis, the general time reversible (GTR) with gamma distribution (G) substitution model was predicted with subset partitions positions 1, 2, and 3 (scheme lnL: 5651.56731 and AIC: 11715.13462) using PartitionFinder version 1.1.1 [22], which was previously used for dataset of grassland caterpillars (Lepidoptera: Lymantriinae: Gynaephora) [26]. In case of Bayesian analysis, optimal partitioning strategy was estimated, which returned three data partitions with two models and two rates. The Bayes block was written to accommodate the partitions, models and rates indicated by PartitionFinder version 1.1.1 as follows: position 1—GTR + G; position 2—GTR +I+ G; position 3—HKY + I + G. The best scheme was chosen based on the score of lnL: -5654.66009 and AIC: 11713.32018. The respective predicted models in each method were subjected to further analysis.

**Fig 5:**
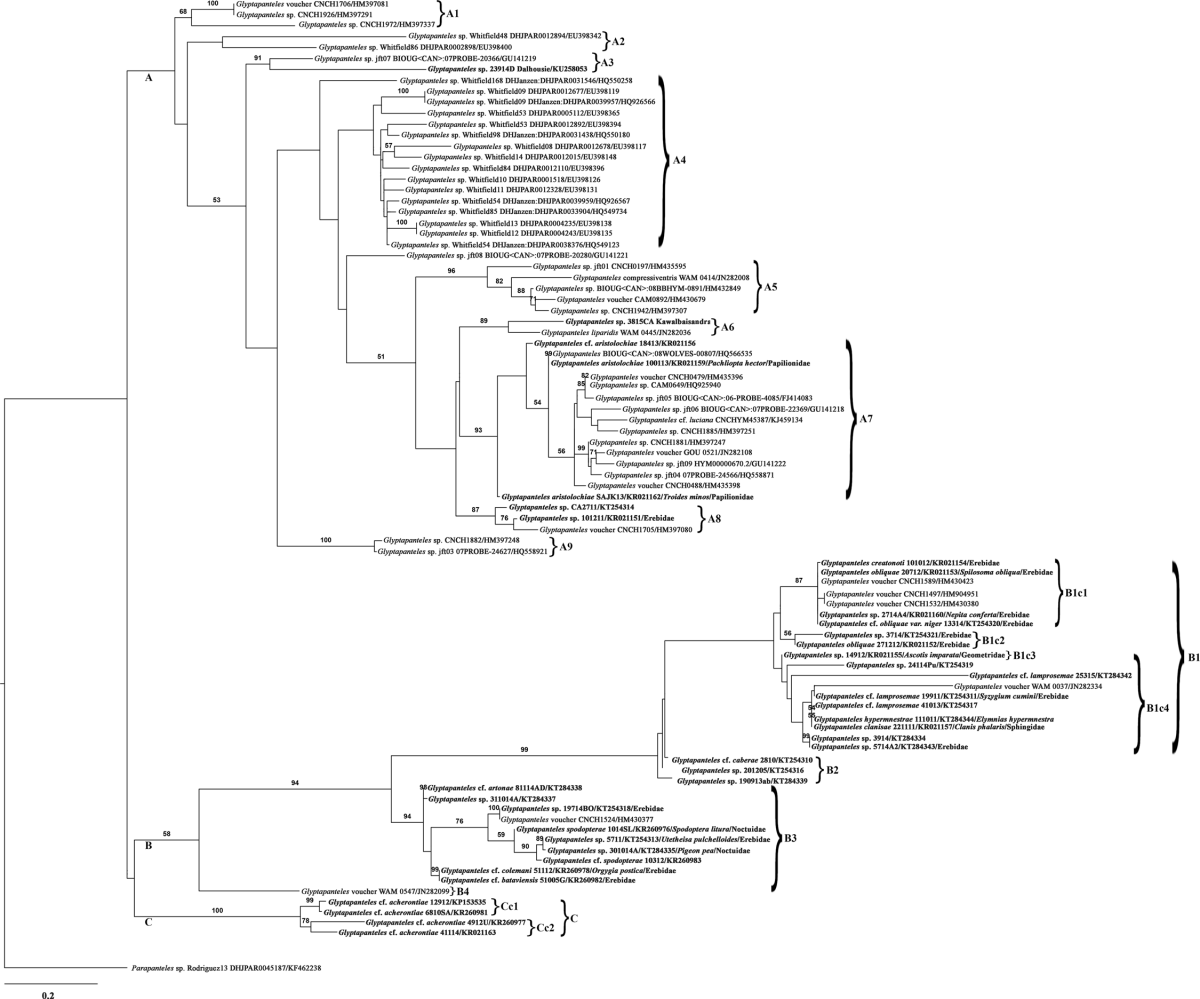
Maximum likelihood phylogenetic tree of partial COI gene sequences of *Glyptapanteles* spp. The scale bar indicates the number of substitutions per nucleotide position. Numbers at nodes represent bootstrap values with 1000 replicates (only values >50 are shown).

**Fig 6:**
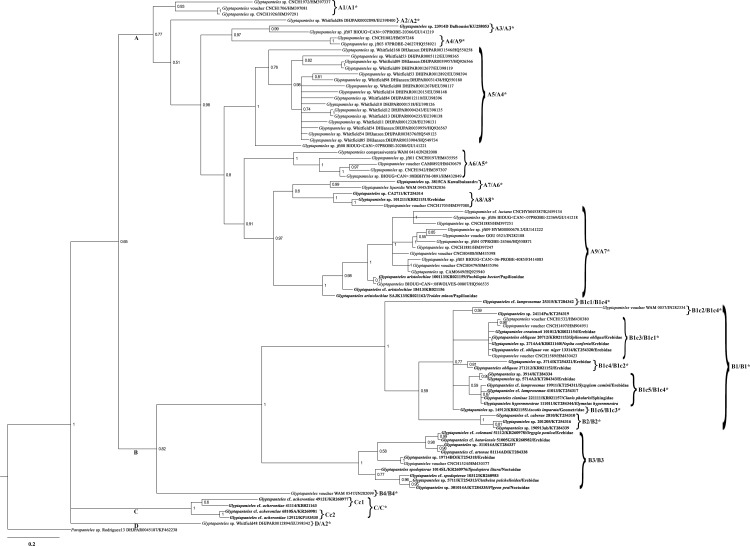
Bayesian inference phylogenetic tree of partial COI gene sequences of *Glyptapanteles* spp. The scale bar indicates the number of substitutions per nucleotide position. Posterior probabilities supporting nodes (>0.50) are shown. The sequences of isolates obtained in the present study are shown in bold and the closest sequences are shown with their GenBank accession number. * Symbol represents the similar ML tree clade number.

Phylogenetic reconstructions based on the COI gene region of *Glyptapanteles* spp. using ML and BI methods yielded trees with nearly similar topologies. Both trees were generated with higher bootstrap value (BV) as well as posterior probability (PP) values. An examination of the phylogenetic trees of ML (Fig 5) and BI (Fig 6) reveals that all *Glyptapanteles* species were resolved into three major clades A, B and C. Clade A composed of *G*. *compressiventris*, *G*. *aristolochiae*, *G*. *liparidis*, *G*. cf. *luciana* and other *Glyptapanteles* spp. (BV>50, PP = 0.77). Clade A was further subdivided into nine subclades wherein Indian species grouped together along with Neotropical and Thailand species in subclade A3, A7, A8 and A9, four of them were well supported (BV ≥87%).

Clade B was subdivided into four major subclades: B1, B2, B3 and B4 with 12 species *viz*. *G*. *creatonoti*, *G*. *obliquae*, *G*. cf. *obliquae* var. niger, *G*. cf. *lamprosemae*, *G*. *hypermnestrae*, *G*. *clanisae*, *G*. cf. *caberae*, *G*. cf. *artonae*, *G*. *spodopterae*, *G*. cf. *spodopterae*, *G*. cf. *colemani*, *G*. cf. *bataviensis* and other *Glyptapanteles* species (BV = 58, PP = 0.82). Clade C contained only one species: *G*. cf. *acherontiae* (BV = 100, PP = 1) and was subdivided into two subclades.

#### 3.4. Evolutionary divergence based on the COI gene

The assessment of COI gene was performed using the evolutionary distances based on intra- and inter-specific species divergences. Kimura’s 2 Parameters model (K2P) is a widely referred method for evaluating marker gene. The computed K2P intra- and inter-specific distances for COI gene are shown in S2 Table. The distribution of genetic divergence revealed that most of the COI pairwise distances were lower in species obtained in same clade as compared with species from distinct clades.

## Discussion

To date, there have been no studies on the Indian Microgastrinae combining morphological, molecular and ecological data. During BLAST analysis of COI gene of *Glyptapanteles* spp., we could not find any representatives of our sequenced species in GenBank database. This is due to the fact that none of the sequences of Indian *Glyptapanteles* were deposited before this study.

Conversely, in the Neotropics, this combined approach has been explored extensively [6, 7, 25, 27, 28]. Previous phylogenies including multiple members of microgastrine genera have shown patterns of high host specificity within species and a high degree of host conservatism among closely related species. Barcoding microgastrine wasps *viz*. *Alphomelon*, *Apanteles*, *Cotesia*, *Dolichogenidea*, *Glyptapanteles*, and *Microplitis* has increased their species richness by more than 70% [27]. COI gene has been considered extensively for parasitoid species delineation [27]. Within the subfamily Microgastrinae it has obtained a high level of accurateness in deciphering species relationships along with other genes [27, 28]. Therefore, in the present study COI gene was chosen for species delineation of Indian *Glyptapanteles* species.

For phylogenetic construction, ML and BI methods were applied successfully on *Glyptapanteles* spp. previously [25] and *Gynaephora* spp. (Lepidoptera: Erebidae: Lymantriinae) [26] to understand relationships across the species and related genera. In order to get more reliability of relationship among the species with two different algorithmic methods, we decided to generate trees using ML and BI methods.

Based on the ML (Fig 5) and BI analysis (Fig 6), we inferred that the majority of the Neotropical species were grouped together in separate clade (A) while most of the Indian species shared a different clade (B). Significant difference in the degree of host specificity combining morphological data was observed in the Indian populations. In ML tree, the Indian species were grouped into 8 well supported clades/ species-groups: A3, A6, A7, A8, B1, B2, B3 and C (Table 3). Perhaps our observations were in line with those mentioned in previous study [29] wherein the combined analysis identified four well-supported clades within *Cotesia* discriminating 25 species. The species-groups delineated in the present study using barcoding and life history data are as follows:

**Table 3:** Species-groups of *Glyptapanteles* in India with total number of species currently within a group. **Lepidoptera host families:** “?” Unknown. **MOR**, **DNA**, **BIO:** degree of group support by morphological (MOR), molecular (DNA), and biological (BIO) data. “**+**” Strong support, “**-**”No support, “**P**” Partial support, “**?**” Unknown.

Species-group	Total number of morphospecies within a group	Lepidoptera host families	MOR	DNA	BIO
A3	1	?	**+**	**+**	?
A6	1	Erebidae	?	**+**	?
A7	2	Papilionidae	**+**	**+**	**+**
A8	2	Erebidae	-	**-**	P
B1 (B1c1, B1c2, B1c3, B1c4)	9	Erebidae, Geometridae, Nymphalidae, Sphingidae	P	P	P
B2	3	Erebidae	-	**+**	?
B3	7	Erebidae, Noctuidae	P	**+**	P
C (Cc1, Cc2)	1	Geometridae	**+**	**+**	**+**

### 4.1 Provisional species-group with distinct morphological and molecular data

Subclade A3 in ML tree (Fig 5) and BI tree (Fig 6), with a high altitude gregarious species (KU258053) with distinct character of first mediotergite strongly narrowed from a wide base to a very narrow apex and about 3.91 times longer than its apical width (extremely unusual with Indian *Glyptapanteles* spp.) shares the clade with a Canadian species (GU141219). This provisional species (KU258053) deserves special attention as according to K2P analysis, pairwise inter-specific K2P distance with the Canadian species (GU14129) is lesser (0.052) than the neighboring Indian species (KR021159) (0.077).

### 4.2 Provisional species-group lacking sound morphological data and biological data

Subclade A6 of the ML tree (equivalent to A7 of BI tree) with a south Indian species (Code 3815CA) sharing the clade with *G*. *liparidis* (JN282036 from Thailand) demands explanation as this pattern remains undetectable using COI gene.

### 4.3 Provisional species-group sharing common host family for Indian species

Subclade A7 of the ML tree (equivalent to A9 of BI tree) with two populations of *G*. *aristolochiae* (KR021159 and KR021162), both parasitizing the same host family Papilionidae but different hosts- *Pachliopta hector* and *Troides minos*, respectively, remain well supported with short pairwise intra-specific K2P distance (0.022). This is due to host conservatism or clade-limited host utilization (CLHU), which refers to closely related parasitoid species in a single clade displaying a strong tendency to attack closely related hosts, within the same family or genus, though prominent within particular portions of the tree [28].

### 4.4 Provisional species-group lacking sound taxonomic explanation

Subclade A8 contains two populations of *Glyptapanteles* spp. (KT254314 and KR021151). The very placement of these two species in clade A demands further studies as morphologically both these species show similarity to *G*. *nigrescens* and *G*. *obliquae*, respectively. Perhaps these species need to be re-examined for additional morphological characters in combination with additional genes.

### 4.5 Provisional species-group partially supported by morphological, molecular and biological data

Based on the ML tree, clade B was subdivided into four major subclades- B1, B2, B3 and B4. Three of them (B1, B2 and B3 which include Indian species) are well supported (bootstrap values ≥94%). Subclade B1 is further divided into four subclades wherein B1c1 and B1c2 show Indian populations of *Glyptapanteles* spp. sharing common host family Erebidae (bootstrap values ≥87% and ≥56% respectively). Primarily all the populations of *G*. *obliquae* and *G*. *creatonoti* and species which show close resemblance to either of them with their hosts from family Erebidae share the same clade. K2P distance between a species from B1c1 (KR021153) and another species from B1c2 (KT254321) is low (0.035) supporting family specific-clusters. One pair of species worth special attention is *G*. *creatonoti* and *G*. *obliquae* (KR021154 and KR021153, respectively). Their sequences differ by only 1bp and they parasitize different host species of Erebidae: Arctiinae. Morphology and ecology expose this pair of species, giving confidence that a single bp difference in the sequences can also reveal a real species-level difference.

In BI tree, interestingly one population of *Glyptapanteles* sp. (KT254319) which is morphologically distinct as compared to other *Glyptapanteles* spp. in having a long ovipositor stands separately forming a separate subclade B1c2.

In ML tree, subclade B1c3 holds a single population *Glyptapanteles* sp. reared from host of the family Geometridae. Also subclade B1c4 does not show any major preference in host specificity (with hosts from Erebidae, Nymphalidae and Sphingidae). In this case, at the family level clade-limited host utilization (CLHU) is dispersed (unlike the case of B1c1 and B1c2 sharing common host family Erebidae). Conversely, wasps that parasitize other families (like in this case Geometridae, Sphingidae and Nymphalidae) form no obvious clusters or patterns (as seen in subclade B1c4). Such cases need additional one or more genes to decipher inter-relationships.

### 4.6 Provisional species-group with COI variation that fails to covary with morphological and biological data.

Similarly, in the subclade B2 (same in ML and BI tree) many morphologically cryptic species, possessing very subtle morphological differences, were grouped together with their hosts either from family Noctuidae or Erebidae. COI gene was not sufficient to distinguish these species.

### 4.7 Provisional species-group with variation in cocoon laying pattern- solitary as well as gregarious

Subclade B3 (same in ML and BI tree)- *Glyptapanteles spodopterae* reared from *Spodoptera litura* (Noctuidae) and *G*. cf. *spodopterae* (also reared from a Noctuid host-

?*Melanephia mosara*), and both solitary in nature, were grouped along with many gregarious species reared from hosts of family Erebidae which needs further studies with alternate genes to resolve. However, significant morphological similarity (all gregarious in nature) is noticed in B3 for KT284337, KT254318, and KT284335 which either could be a single species or very closely related species. Pairwise K2P distance from the neighbouring species of B1c1 (KR021153) and one of these cryptic species from B3 (KT284337) is comparatively high (0.085) and is well supported by morphological differences among both these.

### 4.8 Provisional species-group with strong support based on morphology, molecular and ecological data (possessing single host or same host family with uniform cocoon structure)

In ML tree- subclade C with four populations of *G*. cf. *acherontiae* (all sharing a common morphological character- median area of second tergite very small and noticeably convex, at apex apparently not even wide as apex of first tergite) is grouped further into two subclades: Cc1 having *G*. cf. *acherontiae* from southern India (reared from a Geometridae caterpillar on the same host plant *Melia azedarach* L.) and Cc2 having *G*. cf. *acherontiae* populations from northern and north-eastern India. Clade C showed similar results in ML as well as in BI tree wherein the various populations of *G*. cf. *acherontiae* were grouped into two subclades.

K2P genetic distance between KP153535 (from Cc1) and KR021163 (Cc2) is low (0.024). This shows mild difference due to geographical separation (Cc1 and Cc2 are geographically realistic subset of species). This increasingly expected phenomenon of high host specificity with very narrow host ranges within the parasitoids has been well noticed in earlier studies too [27]. The species-group containing four populations of *G*. cf. *acherontiae* is morphologically distinct, supported with higher bootstrap (100%) as well as higher posterior probability value (1). This species-group is also remarkably separated from others based on host family record- Geometridae and distinct cocoon laying pattern (compact congregated mass of white cocoons noticed uniformly in all the populations). Also pairwise inter- specific K2P distance from *G*. *obliquae* reared from *Spilosoma obliqua* (Erebidae) in neighboring subclade B1c1 is high (0.135).

## Conclusion

This is the first phylogenetic study to resolve a diverse and geographically realistic subset of species within the genus *Glyptapanteles* to correlate the host specialization in India. Although some relationships were resolved, an additional gene/genes evolving more slowly than COI, but fast enough to separate closely related groups of species, may provide the extra resolution required to completely resolve the Indian *Glyptapanteles*. Furthermore, it is necessary to incorporate these findings into a global context by adding additional geographic and economically important subsets of *Glyptapanteles* and to describe the Indian species delineated in this work. Wasp diversity, and likewise diversity of host species they parasitize, makes them particularly relevant in the control of lepidopteran populations within an explosively diverse ecosystem.

## Supporting Information

S1 Table Details of blastn hits obtained for COI sequences of *Glyptapanteles* spp. Blastn hits for COI sequences.
(XLSX)

S2 Table Estimates of evolutionary divergence between sequences. K2P intra- and inter-specific distances for COI gene.
(XLS)

## References

[1] Blanchard EE. Microgastrinos argentinos, nuevos y poco conocidos. Segunda parte. Physis. Revista de la Sociedad Argentina de Ciencias Naturales. 1936; 12: 137–152.

[2] Muesebeck CFW. New Neotropical wasps of the family Braconidae (Hymenoptera) in the U.S. National Museum. Proceedings of the United States National Museum. 1958; 107: 405–461.

[3] Mason WRM. The polyphyletic nature of *Apanteles* Foerster (Hymenoptera: Braconidae): a phylogeny and reclassification of Microgastrinae. Memoirs of the Entomological Society of Canada. 1981; 115: 1–147. 10.4039/entm113115fv

[4] Whitfield JB, Benzing A, Ponce F. Review of the *Glyptapanteles* species (Hymenoptera: Braconidae, Microgastrinae) attacking noctuids in field crops in the Neotropical Region, with description of two new species from the Ecuadorian Andes. Journal of Hymenoptera Research. 2002; 11: 152–165.

[5] Arias-Penna DC. Phylogenetics, taxonomy and host use of Neotropical *Glyptapanteles* parasitoid wasps. Entomological Society of America 61th, Annual Meeting. Ten-Minute Papers, SysEB Section: Systematics of Hymenoptera. Austin, TX, United States, 2013.

[6] Fernandez-Triana JL, Whitfield JB, Rodriguez JJ, Smith MA, Janzen DH, Hallwachs W, et al Review of *Apanteles sensu stricto* (Hymenoptera, Braconidae, Microgastrinae) from Area de Conservacion Guanacaste, northwestern Costa Rica, with keys to all described species from Mesoamerica. ZooKeys. 2014; 383: 1–565. 10.3897/zookeys.383.6418 24624021 PMC3950464

[7] Arias-Penna DC. The Neotropics is teeming with little parasitoid wasps: *Glyptapanteles*. Hamuli. 2014; 5: 1–3.

[8] Whitfield JB, Rodriguez JJ, Masonick PK. Reared microgastrine wasps (Hymenoptera: Braconidae) from Yanayacu Biological Station and environs (Napo Province, Ecuador): Diversity and host specialization. Journal of Insect Science. 2009; 9: 1–22. 10.1673/031.009.3101 PMC3011880 19613864

[9] Arias-Penna DC. Approximation to classification and diversity of *Glyptapanteles* (Braconidae, Microgastrinae) from the Neotropics based on material from northwestern Costa Rica. Entomological Society of America 59th, Annual Meeting. Graduate Student Ten-Minute Paper Competition. Reno, NV, United States, 2011.

[10] Wilkinson DS. A revision of the Indo-Australian species of the genus *Apanteles* (Hym. Bracon.). Part II. Bulletin of Entomological Reserach. 1928; 19: 109–146.

[11] Nixon GEJ. A reclassification of the tribe Microgasterini (Hymenoptera: Braconidae). Bulletin of Bristish Museum of Natural History Entomology series, Supplement 2 1965; 1–284.

[12] Gupta A, Pereira B. A new species of *Glyptapanteles* (Hymenoptera: Braconidae: Microgastrinae), a larval parasitoid of *Elymnias hypermnestra* (Linnaeus) (Lepidoptera: Nymphalidae), along with some new host records of parasitoids from Peninsular India. Zootaxa. 2012; 3227: 54–63.

[13] Gupta A. Three new species of reared parasitic wasps (Hymenoptera: Braconidae: Microgastrinae) from India. Zootaxa. 2013; 3701: 365–380. 10.11646/zootaxa.3701.3.6 26191590

[14] Gupta A, Fernández-Triana JL. Diversity, host association, and cocoon variability of reared Indian Microgastrinae (Hymenoptera: Braconidae). Zootaxa. 2014; 3800: 1–101. 10.11646/zootaxa.3800.1.1 24870869

[15] Yu DSK, van Achterberg C, Horstmann K. Taxapad 2012, Ichneumonoidea 2011. Database on flash-drive. www.taxapad.com, Ottawa, Ontario, Canada.

[16] Rugman-Jones PF, Hoddle MS, Stouthamer R. Nuclear-mitochondrial barcoding exposes the global pest western flower thrips (Thysanoptera: Thripidae) as two sympatric cryptic species in it’s native California. Journal of Economic Entomology. 2010; 103: 877–886. 20568635 10.1603/ec09300

[17] Benson DA, Karsch-Mizrachi, Lipman DJ, Ostell J, David L. Wheeler Nucleic Acids Res. 2005 1 1; 33(Database issue): D34–D38. Published online 2004 December 17. 15608212 10.1093/nar/gki063 PMC540017

[18] Altschul SF, Gish W, Miller W, Myers EW, Lipman DJ. Basic local alignment search tool. Journal of Molecular Biology. 1990; 215: 403–410. 2231712 10.1016/S0022-2836(05)80360-2

[19] Edgar RC. MUSCLE: multiple sequence alignment with high accuracy and high throughput, Nucleic Acids Research. 2004; 32: 1792–97. 15034147 10.1093/nar/gkh340 PMC390337

[20] Tamura K, Stecher G, Peterson D, Filipski A, Kumar S. MEGA6: Molecular Evolutionary Genetics Analysis version 6.0. Molecular Biology and Evolution. 2013; 30: 2725–2729. 10.1093/molbev/mst197 24132122 PMC3840312

[21] Lanfear R, Calcott B, Simon Y, Ho W, Stephane GS. PartitionFinder: Combined Selection of Partitioning Schemes and Substitution Models for Phylogenetic Analyses. Molecular Biology and Evolution 2012; 29: 1695–1701. 10.1093/molbev/mss020 22319168

[22] Ronquist F, Teslenko M, van der Mark P, Ayres DL, Darling A, Höhna S, et al MrBayes 3.2: efficient Bayesian phylogenetic inference and model choice across a large model space. Systematic Biology. 2012; 61: 539–542. 10.1093/sysbio/sys029 22357727 PMC3329765

[23] Stamatakis A. RAxML-VI-HPC: maximum likelihood-based phylogenetic analyses with thousands of taxa and mixed models. Bioinformatics. 2006; 22: 2688–2690. 16928733 10.1093/bioinformatics/btl446

[24] Kimura M. A simple method for estimating evolutionary rate of base substitutions through comparative studies of nucleotide sequences. Journal of Molecular Evolution. 1980; 16: 111–120. 7463489 10.1007/BF01731581

[25] Whitfield JB, Mardulyn P, Austin AD, Dowton M. Phylogenetic relationships among microgastrine braconid wasp genera based on data from the 16S, COI and 28S genes and morphology. Systematic Entomology. 2002; 27: 337–359. 10.1046/j.1365-3113.2002.00183.x

[26] Yuan M-L, Zhang Q-L, Wang Z-F, Guo Z-L, Bao G-S. Molecular Phylogeny of Grassland Caterpillars (Lepidoptera: Lymantriinae: *Gynaephora*) Endemic to the Qinghai-Tibetan Plateau. López-Vaamonde C, ed. PLOS One. 2015; 10(6): e0127257 10.1371/journal.pone.0127257 26053874 PMC4459697

[27] Smith AM, Rodriguez JJ, Whitfield JB, Deans AR, Janzen DH, Hallwachs W, et al Extreme diversity of tropical parasitoid wasps exposed by iterative integration of natural history, DNA barcoding, morphology, and collections. Proceedings of the National Academy of Sciences, USA. 2008; 105: 12359. 10.1073/pnas.0805319105 PMC2518452 18716001

[28] O’connor JM. Phylogenetic patterns of host specialization in two tropical Microgastrinae (Hymenoptera: Braconidae) parasitoid wasp genera. PhD Thesis, University of Illinois, Urbana-Champaign, 2011.

[29] Salzat-Michel A, Whitfield JB. Preliminary evolutionary relationships within the parasitoid wasp genus *Cotesia* (Hymenoptera: Braconidae: Microgastrinae): combined analysis of four genes. Systematic Entomology. 2004; 29: 371–382.

